# Medication adherence in patients with cluster headache and migraine: an online survey

**DOI:** 10.1038/s41598-023-30854-y

**Published:** 2023-03-20

**Authors:** Florian Rimmele, Britta Müller, Nadine Becker-Hingst, Sophia Wegener, Stefanie Rimmele, Peter Kropp, Tim P. Jürgens

**Affiliations:** 1 Department of Neurology, University Medical Center Rostock, University of Rostock, Gehlsheimer Str. 20, 18147 Rostock, Germany; 2 Institute of Medical Psychology and Medical Sociology, University Medical Center Rostock, Gehlsheimer Str. 20, 18147 Rostock, Germany; 3 Headache Center North-East, University Medical Center Rostock, Gehlsheimer Str. 20, 18147 Rostock, Germany; 4 Institut für Pädagogische Psychologie “Rosa und David Katz”, University Rostock, August-Bebel-Str. 28, 18051 Rostock, Germany; 5 Berufsförderungswerk Stralsund GmbH, Große Parower Straße 133, 18435 Stralsund, Germany; 6 Department of Anaesthesia, Krankenhaus Buchholz, Steinbecker Str. 44, 21244 Buchholz, Germany

**Keywords:** Neuroscience, Neurology

## Abstract

To examine factors for adherent and non-adherent behavior in patients with cluster headache and migraine. Adults with cluster headache or migraine were included in this anonymous online survey using a questionnaire accessed via homepages of headache support groups. Medication adherence in preventive treatment was measured with the Medication Adherence Report Scale (MARS-D). Factors for non-adherent behavior were examined (subjective socioeconomic status, psychological comorbidities, self-efficacy, coping, side effects, expectations of treatment, information on medical treatment, and trust in the physician/treatment concept). 200 participants (n = 58 with cluster headache, n = 142 with migraine) were included. The rate of medication adherence in preventive treatment was 32.8% for participants with cluster headache and 20.4% for migraine. The most common reasons for low adherence in participants with cluster headache were altering the prescribed medication dose (34%) or taking less than instructed (14%), which was mostly due to insufficient benefit from the medication or side effects. Positive expectations of medical treatment (*p* ≤ 0.05) correlated significantly with adherent behavior in cluster headache. Furthermore, the adherence-promoting factors coping and self-efficacy were more pronounced in patients with cluster headache than in those with migraine (*p* < 0.05). This study is the first to comprehensively investigate medication adherence and factors influencing adherent/non-adherent behavior in patients with cluster headache. Patients with cluster headache had similar adherence levels to patients with migraine, but had higher resources of adherence-promoting factors.

## Introduction

Cluster headache (CH) is amongst the most severe and disabling painful conditions^1,2^. CH is also called "suicide headache" because the pain intensity and impairment of quality of life can be so severe that sufferers tend to have suicidal thoughts and tendencies^3^. It is further associated with psychiatric comorbidities such as depression, anxiety, or aggressive behavior^3,4^, especially in chronic CH. Diagnosis of chronic CH requires cluster attacks occurring for one year or longer without remission, or with remission periods lasting less than 3 months and therefore often represent an even greater burden for the patient than episodic CH^1^. This results in high direct and indirect costs for both the affected patients and the healthcare system, especially in chronic cases^5^. The main focus of treating CH is on acute suppression of attacks through medication and a prophylactic treatment with daily intake to reduce attack frequency^6^.

A pivotal factor for successful treatment is high adherence^7^. This concept of treatment compliance is defined as the extent to which a person's behavior—taking medication, following a therapeutic concept or changing one’s lifestyle—is in line with the agreed recommendations of a healthcare provider^8^. Due to the multiple interactions between treatment provider, patient and the healthcare system, adherence is a complex construct that is influenced by multiple factors. The World Health Organization (WHO) distinguishes between the following factors: patient-related factors (e.g. self-efficacy), health system-related factors (e.g. trust in the doctor and in the treatment), therapy-related factors (e.g. strong burden of side effects), condition-related factors (e.g. comorbidities) and socioeconomic factors (e.g. low socioeconomic status)^8^. Whereby self-efficacy is the personal conviction of people to be able to cope with difficult demands by their own efforts^9^. Self-efficacy expectations arise from confidence in one's own ability to act and describe a person's expectation that they can consciously influence themselves and things in the world. Self-efficacy expectations contribute to the activation and maintenance of health-relevant behaviour and are positively related to the development of adherent behaviour^10^.

Non-adherent behavior is a great challenge in providing efficient medical care. It has even been described as the “Achilles’ heel of modern medicine”, and leads to higher morbidity, mortality, unnecessary utilization of healthcare resources^11^. 25–50% of headache patients are non-adherent to preventive medication^12^. In patients with chronic headache adherence to prescribed medication treatments is even lower, with non-adherence rate of 50–60%^11^. However, no patients with CH were studied in these trials. Medication adherence is a particularly important aspect in CH, as medication over a long period of time is the mainstay of clinical treatment in this severe pain disorder.

While adherence in the treatment of migraine is already relatively well explored^13–16^, studies of adherence in the treatment of CH are rare. The aim of the present study was to examine the hypothesis that adherence in patients with CH differs from that in migraine and that there are different factors for adherent and non-adherent behaviour in patients with CH versus migraine.

## Methods

### Participants

The present study included participants aged 18 years and older with a medical history of cluster headache or migraine with or without aura. Diagnoses were based on participants’ self-report at the beginning of an anonymous online survey with a browser-based questionnaire, whose link was provided on the homepages of headache support groups. The online questionnaire took about 10 min to complete. All study participants were provided with written information about the study procedure prior to inclusion. Inclusion was only possible if participants provided informed consent, which was confirmed by sending the data at the end of the survey. The data were recorded anonymously and documented using EvaSys (Education Survey Automation Suite), a software package for online surveys of the IT Center of the University of Rostock.

### Measures

To assess medication adherence and factors included in the WHO report^8^ known to influence adherent behavior, we developed a comprehensive questionnaire consisting of standardized self-rating procedures and items constructed for the purpose of this investigation.

Medication adherence was measured with the German version of the Medication Adherence Report Scale (MARS-D), which consists of five items. A score of 25 indicates maximum adherent behavior. The psychometric properties of the MARS-D have been tested in two study populations, and it is the questionnaire with the best psychometric properties for the German-speaking area^17,18^.

In total, eight factors for non-adherent behavior were examined: Subjective socioeconomic status was measured with the German version of the MacArthur Scale^19^.To assess mental comorbidities, the Prime MD Patient Health Questionnaire (PHQ) in its German version (PHQ-D) was used^20^. Self-management skills like self-efficacy and coping are important factors for adherence. To assess these factors, we used the Questionnaire for the Assessment of Resources and Self-Management Skills (FERUS) which contains 66 items allocated to the following seven scales: motivation to change, coping, self-observation, self-efficacy, self-verbalization, hope, and social support^21^. As there were no suitable questionnaires for further examined factors on non-adherent behavior (like side effects, expectations of the treatment, information on medical treatment, and trust in the doctor and the treatment concept) these factors were measured using items constructed for the purpose of the present survey, with items rated on a 5-point Likert scale from “always” to “never” (See Supplementary Table 1).

### Analyses

The data were analyzed using the statistical program SPSS (version 23.0). The main variables were analyzed using descriptive statistics. Pearson's Phi correlation (between two dichotomous variables) and Eta correlation (between dichotomous and metric variables) were used to measure the strength of association.

To compare means between two independent samples, under the assumption of a normal distribution, t-tests were used. In the case of non-normal distribution, Mann–Whitney U tests were applied. As this was an exploratory study results were not corrected for multiple testing.

### Ethical approval and informed consent

This online survey was approved by the Ethics committee of the University Medical Center Rostock (A2016-0051). Written informed consent was obtained from all participants.

## Results

**Figure 1:**
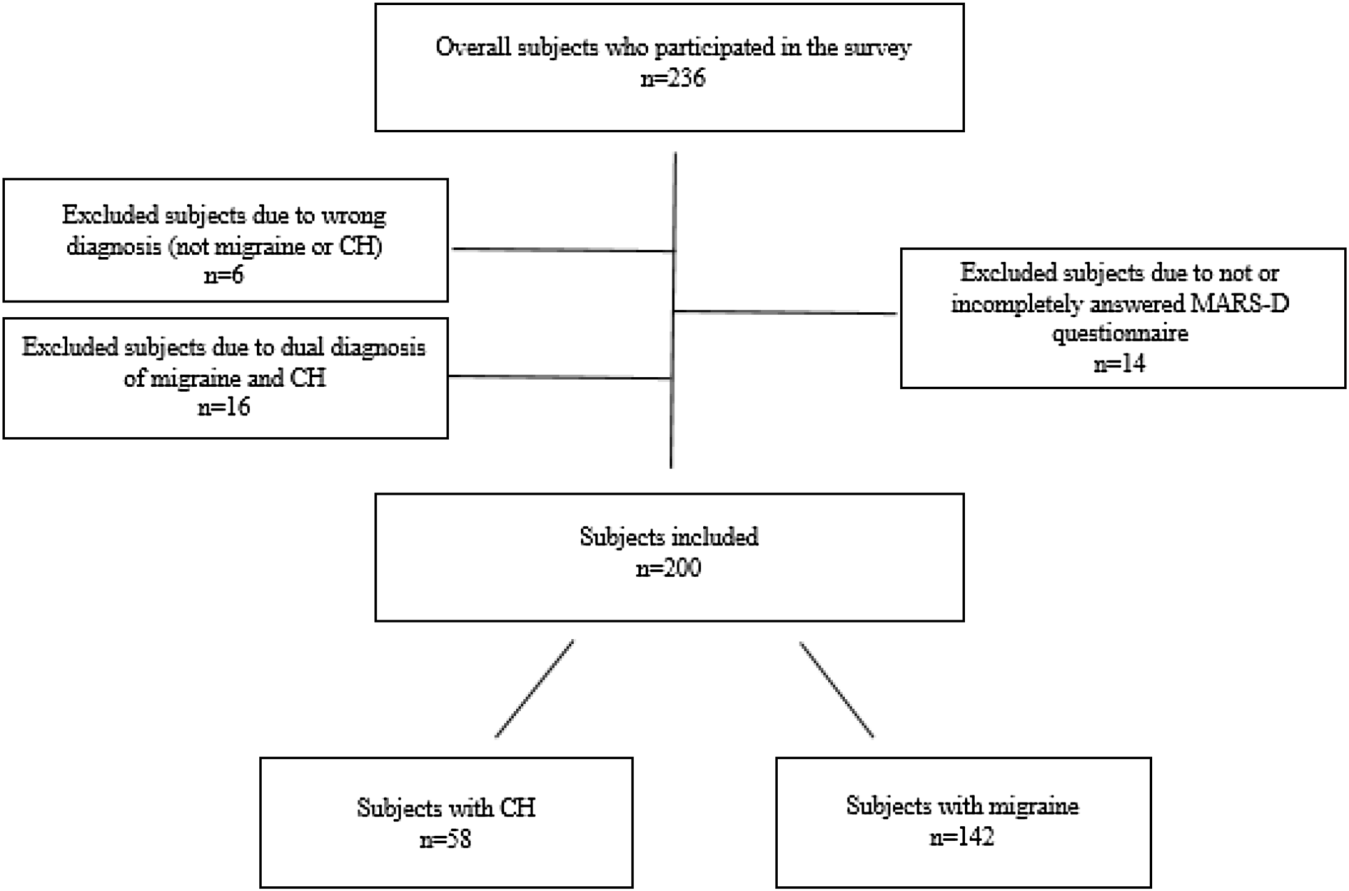
Flowchart for the inclusion of the subjects in the study.

From the beginning of May 2016 to the middle of June 2016, n = 200 patients took part in the survey, of whom n = 58 were allocated to group 1, with the medical diagnosis of cluster headache, and n = 142 to group 2, with the medical diagnosis of migraine with or without aura (see Table 1). For the inclusion of the patients in the study, see the flowchart (Fig. 1). The patients with cluster headache had been suffering for an average of 15.8 years (SD = 11.1). The medical diagnosis had been made 8.4 years ago (SD = 7.9), and treatment had begun 8.6 years ago (SD = 8.3). The patients with migraine had been suffering for an average of 22.8 years (SD = 13.3), the medical diagnosis had been made 15.9 years ago (SD = 12.4) and medical treatment had begun 13.1 years ago (SD = 11.4). The majority of patients in both groups were severely impaired (Supplementary Table 2a,b).

**Table 1:** Demographics and characteristics of the sample.

	Overall sample n = 200	Cluster headache n = 58	Migraine n = 142	*p*
Age (y), mean, [SD] (Missing n = 3)	43.31 [11.92]	44.71 [11.25]	42.76 [12.17]	0.285^a^
Sex (%)				**< 0.001**^**b**^
Female	151 (76.3)	17 (29.8)	134 (95.0)	
Male (Missing n = 2)	47 (23.7)	40 (70.2)	7 (5.0)	
Disease duration, mean, [SD] (missing n = 1)	20.77 [13.06]	15.78 [11.09]	22.83 [13.28]	**< 0.001**^**a**^
Duration since diagnosis was made, mean, [SD] (missing n = 1)	13.76 [11.75]	8.41 [7.88]	15.94 [12.38]	**< 0.001**^**a**^
Duration since the beginning of the preventive therapy, mean, [SD] (missing n = 8)	11.81 [10.80]	8.57 [8.29]	13.14 [11.43]	**0.002**^**a**^
Marital status (%)				0.592^b^
Married	96 (48.0)	29 (50.0)	67 (47.2)	
Single	42 (21.0)	13 (22.4)	29 (20.4)	
Divorced	15 (7.5)	3 (5.2)	12 (8.5)	
Widowed	1 (0.5)	1 (1.7)	0 (0.0)	
Partnership	45 (22.5)	12 (20.7)	33 (23.2)	
Civil union (missing n = 0)	1 (0.5)	1 (1.6)	1 (0.7)	
Education (%)				0.102^b^
No school-leaving certificate	1 (0.5)	0 (0.0)	1 (0.7)	
Elementary	9 (4.5)	4 (7.0)	5 (3.5)	
Secondary	86 (43.0)	28 (49.1)	58 (40.8)	
Fachhochschulreife	24 (12.0)	10 (17.5)	14 (9.9)	
Baccalaureate (missing n = 1)	79 (39.5)	15 (25.9)	64 (45.1)	
Professional qualification (%)				0.217^b^
No professional qualification	10 (5.1)	2 (3.5)	8 (5.7)	
Other professional qualification	2 (1.0)	1 (1.7)	1 (0.7)	
Professional-vocational training	51 (25.5)	17 (29.3)	34 (24.3)	
Professional-school-based training	38 (19.0)	9 (15.5)	29 (20.7)	
Technical college	31 (15.5)	13 (22.4)	18 (12.9)	
Advanced technical college	22 (11.0)	8 (13.8)	14 (10.0)	
University (missing n = 3)	43 (21.5)	7 (12.1)	36 (25.7)	
Employment (%)				0.228^b^
Not in employment	31 (15.5)	8 (13.8)	23 (16.2)	
Seeking employment	12 (6.0)	5 (8.6)	7 (4.9)	
Disability pension	13 (6.5)	6 (10.3)	7 (4.9)	
Full-time	91 (45.5)	30 (51.7)	61 (43.0)	
Part-time	43 (21.5)	8 (13.8)	35 (24.6)	
Training	5 (2.5)	1 (1.7)	4 (2.8)	
Other leave of absence (missing n = 0)	5 (2.5)	0 (0.0)	5 (3.5)	

^a^Welch test, ^b^Pearson's chi-squared test.

Significant values are in bold.

### Rate of medication adherence

The rate of medication adherence using the MARS-D scale, reached a mean score of 22.4 (SD = 2.9) in the group of CH patients and 21.9 (SD = 3.0) in the group of migraine patients. In line with the literature, the cut-off for adherence was set strictly at a score of 25, and a score ≤ 24 was accordingly rated as non-adherent behavior^22,23^. This resulted in an adherence rate of 32.8% for patients with cluster headache and 22.0% for migraine patients (*p* = 0.112). Adherence to general medication was assessed with the MARS-D test. No distinction was made between acute pain medication and prophylactic medication.

The individual behavioral aspects contributing either to adherent or non-adherent behavior are evident from the various items of the MARS-D. Tables 2, 3, 4 and 5 presents the distribution of response frequencies for the groups of patients with CH and migraine. None of the patients with CH reported forgetting to take their medication “always” or “often”. Only 8.6% of the patients “sometimes” forgot to take their medication, and 91.4% “never” or “rarely” forgot to do so. Likewise, patients also reported “rarely” or “never” purposely skipping medication (93.1%) or reducing the dose (86.2%). Patients were most likely to report altering the dose, with only 65.5% of patients indicating that they “never” or “rarely” do so. The group of migraine patients showed similar behavior. An item-wise comparison between participants with CH and migraine showed no statistically significant differences for the percentages of responses for all 5 items of the MARS-D.

**Table 2:** Distribution of the response frequencies of the Medication Adherence Report Scale (MARS-D) for patients with cluster headache (n = 58).

MARS-D statement	“always” n(%)	“often” n(%)	“sometimes” n(%)	“rarely” n(%)	“never” n(%)
I forget to take them (missing n = 0)	0 (0.0)	0 (0.0)	5 (8.6)	17 (29.3)	36 (62.1)
I alter the dose (missing n = 0)	2 (3.4)	4 (6.9)	14 (24.1)	8 (13.8)	30 (51.7)
I stop taking them for a while (missing n = 0)	0 (0.0)	0 (0.0)	8 (13.8)	11 (19.0)	39 (67.2)
I decide to miss out a dose (missing n = 0)	0 (0.0)	1 (1.7)	3 (5.2)	5 (8.6)	49 (84.5)
I take less than instructed (missing n = 0)	2 (3.4)	1 (1.7)	5 (8.6)	7 (12.1)	43 (74.1)

**Table 3:** Distribution of the response frequencies of the Medication Adherence Report Scale (MARS-D) in migraine patients (n = 142).

MARS-D statement	“always” n(%)	“often” n(%)	“sometimes” n(%)	“rarely” n(%)	“never” n(%)
I forget to take them (missing n = 0)	1 (0.7)	0 (0.0)	14 (9.9)	48 (33.8)	79 (55.6)
I alter the dose (missing n = 0)	1 (0.7)	9 (6.3)	25 (17.6)	33 (23.2)	74 (52.1)
I stop taking them for a while (missing n = 0)	2 (1.4)	4 (2.8)	17 (12.0)	34 (23.9)	85 (59.9)
I decide to miss out a dose (missing n = 0)	2 (1.4)	5 (3.5)	9 (6.3)	24 (16.9)	102 (71.8)
I take less than instructed (missing n = 0)	2 (1.4)	5 (3.5)	19 (13.4)	30 (21.1)	86 (60.6)

**Table 4:** Mean and standard deviation of individual MARS-D items and sum for both headache types.

MARS-D statement	Cluster headache	Migraine	*p*^a^
I forget to take them (mean, [SD])	4.53 [0.65]	4.44 [0.73]	0.356
I alter the dose (mean, [SD])	4.03 [1.17]	4.20 [0.99]	0.354
I stop taking them for a while (mean, [SD])	4.53 [0.73]	4.38 [0.90]	0.210
I decide to miss out a dose (mean, [SD])	4.76 [0.63]	4.54 [0.87]	0.052
I take less than instructed (mean, [SD])	4.52 [0.98]	4.36 [0.94]	0.297
Sum value (mean, [SD])	22.38 [2.90]	21.92 [3.04]	0.314

^a^Welch test.

**Table 5:** Patients with maximum MARS-D point score.

MARS-D score	Cluster headache (n = 48)	Migraine (n = 142)	*p*
Patients with score 25 n(%)	19 (32.8) (missing n = 0)	31 (22.0) (missing n = 1)	0.112^a^

^a^Pearson's chi-squared test.

### Reasons for stopping the medication

**Figure 2:**
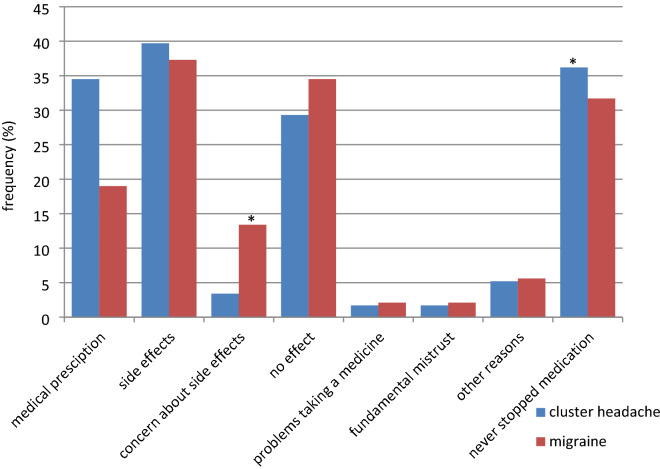
Reasons for stopping the medication in percent; * p ≤ 0.05.

In the questionnaire, CH and migraine patients were asked whether they had ever stopped taking prescribed medication for headache treatment, and if so for what reason. Multiple responses could be given. Figure 2 shows that in total, 36.2% of the CH patients and 31.7% of the migraine patients indicated that they had never stopped taking prescribed medication (*p* = 0.027). Another reason given significantly more often by migraine patients than by CH patients was "concern about side effects" (*p* = 0.042). The most frequently mentioned reason for stopping medication, regardless of medical prescription, was "side effects" and the second most frequent was "no effect" of the therapy in both groups (Fig. 2).

### Medications in cluster headache patients

In both patient groups, the patients who were taking regular medication for headache prevention at the time of the survey were asked which medications they were taking, whether they were able to tolerate these medications, and whether they were effective. Thirty-eight CH patients were regularly taking medication for preventive purposes at the time of the survey. Table 6 demonstrates that the majority (71.1%) were taking verapamil. Moreover, tolerability and effectiveness were also most frequently reported for verapamil (85.2% and 70.4%, respectively).

**Table 6:** Taking prophylactic medication for cluster headache at the time of the survey (n = 38).

Prophylactic medication		Tolerated n(%)	Effective n(%)
Verapamil	27 (71.1)	23 (85.2)	19 (70.4)
Lithium	5 (13.2)	3 (60.0)	4 (80.0)
Topiramate	8 (21.1)	6 (75.0)	6 (75.0)
Other medicines (e.g. cortisone, triptans, methysergide, i.a.)	22 (57.9)	13 (59.1)	11 (50.0)

### Factors for non-adherent behavior

We examined the association between adherence and possible factors for non-adherent behavior based on the WHO classification. For this purpose, correlations between the level of adherence (MARS-D) and the factors subjective socioeconomic status (MacArthur Scale), mental comorbidities (PHQ), self-efficacy (FERUS), coping (FERUS), side effects, expectations of the treatment, information on medical treatment, trust in the doctor and the treatment concept, keeping medical appointments and impairment were assessed. Table 7 depicts the strength of correlations (*r*) and the significance (*p*) between adherence (yes/no) and the possible factors for non-adherent behavior for CH and migraine patients respectively.

**Table 7:** Correlation coefficient r between adherence (yes/no) and sociodemographic and headache-related factors, differentiated by type of headache; *p ≤ 0.05; **p ≤ 0.01; ***p ≤ 0.001.

	Cluster headache	Migraine
Age	0.08^a^	0.09^a^
Sex	− 0.22^b^	0.11^b^
Headache duration (in years)	0.10^a^	0.02^a^
HIT-6 (4 groups)	0.33^b^	0.20^b^
Subjective socioeconomic status	0.14^a^	0.04^a^
Major depression	− 0.02^b^	− 0.04^b^
Self-efficacy	0.08^a^	0.08^a^
Coping	0.19^a^	0.02*^a^
Side effects	0.04^a^	0.02^a^
Expectations of the therapy	0.40*^a^	0.15^a^
Information on medical treatment	0.14^a^	0.13^a^
Trust in doctor and treatment concept	0.16^a^	0.17*^a^
Keeping medical appointments	0.13^a^	0.14^a^

^a^Eta-coefficient, ^b^Phi-coefficient.

Significant values are in bold.

In the group of migraine patients there was a statistically significant correlation between adherence and "Trust in doctor and treatment concept" with a small effect size (Cohen’s d = 0.29). With a Cohen’s d = 0.09, the difference between adherent and non-adherent migraine patients regarding “Coping” is negligible, even though it is statistically significant. In the group of CH patients there was a statistically significant correlation for adherence with “expectations on the therapy” with a large effect size (Cohen’s d = 0.82) (Table 7).

In the group comparison, statistically significant higher rates of self-efficacy (*p* = 0.005) and coping (*p* = 0.028) were found in the CH patients compared to the migraine patients (Table 8). For mental comorbidities, there was no statistically significant difference between the groups, nor between the subgroups of adherent patients (Table 9).

**Table 8:** FERUS results for both headache types.

FERUS item	Cluster headache (n = 58)	Migraine (n = 142)	*p*^a^
Coping, sum value (mean, [SD])	42.24 [6.50] (missing n = 0)	39.70 [7.69] (missing n = 0)	**0.028**
Coping, mean value (mean, [SD])	3.52 [0.54] (missing n = 0)	3.31 [0.64] (missing n = 0)	**0.028**
Self-efficacy, sum value (mean, [SD])	33.21 [4.83] (missing n = 0)	30.45 [6.65] (missing n = 0)	**0.005**
Self-efficacy, mean value (mean, [SD])	3.69 [0.54] (missing n = 0)	3.38 [0.73] (missing n = 0)	**0.005**

^a^Welch test.

Significant values are in bold.

**Table 9:** PHQ-9 results for both headache types.

	Cluster headache (n = 58)	Migraine (n = 142)	*p*^a^
Sum value (mean, [SD])	11.02 [5.11] (missing n = 1)	10.18 [5.45] (missing n = 3)	0.321

Depression level	Cluster headache (n = 58)	Migraine (n = 142)	*p*^b^
No or minimal depression symptoms n(%)	4 (7.0)	17 (12.2)	0.6101
Mild depression symptoms n(%)	20 (35.1)	58 (41.7)	
Moderate depression symptoms n(%)	19 (33.3)	36 (25.9)	
Moderately severe depression symptoms n(%)	10 (17.5)	19 (13.7)	
Severe depression symptoms n(%)	4 (7.0) (missing n = 1)	9 (6.5) (missing n = 3)	

^a^Welch test.

^b^Pearson's chi-squared test.

### Comparison of patients with chronic and episodic cluster headache

In the present survey, 24 patients (41.4%) reported suffering from chronic CH and 34 patients (58.6%) from episodic CH. Adherence as measured with the MARS-D was comparable between both groups (chronic CH: mean = 21.58, SD = 3.41; episodic CH: mean = 22.94; SD = 2.37).

## Discussion

This study is the first study to comprehensively investigate medication adherence and factors influencing adherent/non-adherent behaviour in patients with CH in comparison to another disabling primary headache, migraine. Adherence, as a concept of treatment fidelity and mutual co-operation between treatment provider and patient, is a complex construct which is subject to the influence of multiple factors. With the present survey, we first examined the level of adherence, and investigated factors which may influence non-adherent behavior. The results show that the adherence rate is numerically higher in CH patients than in those with migraine, however, the difference did not reach statistical significance (33% vs. 22%). We were also able to show that factors such as "positive expectations of medical treatment" are significantly correlated with adherent behaviour in CH patients, while "coping" and "trust in doctor and treatment concept" are significantly associated with adherent behaviour in migraine patients.

It is known from the literature that migraine patients show low adherence and persistence (i.e. the time for which a patient takes a prescribed medication) for prophylactic migraine medication^15,24^. The reasons for low adherence can be attributed to altering the prescribed medication dose, or temporarily stopping medication; in contrast, forgetting to take medication did not constitute a relevant reason (Tables 2, 3, 4 and 5). On a critical note, it should be mentioned that in line with the literature^22,23^, we chose a conservative and strict definition of non-adherent behavior with MARS-D scores of ≤ 24. However, in the literature, there are also several studies which set less restrictive cut-off scores of ≥ 23^25^ or > 23^26^ for adherent behavior. To overcome the potential limitations of a strict definition of adherence, we conducted additional analyses using a more permissive approach, the adherence sum score. For this, we examined the sum score of the individual MARS-D items without finding statistically significant differences for the two headache groups (Table 4).

Although no statistically significant difference could be found for the level of adherence between CH and migraine it remains intriguing that a highly burdensome and impairing disorder like CH would imply higher adherence to medical treatment. However, impairment as measured with the HIT-6 did not exert a statistically relevant influence on treatment adherence, neither in CH nor migraine. A higher degree of coping and self-efficacy could be identified as a pivotal factor of adherence in CH patients.

A correlation analysis of the socio-demographic and headache-related factors with the adherence sum score also showed no significant differences compared to the correlation analysis of the cut-off adherence score.

The most frequent reason for skipping or stopping medication mentioned in both groups was "side effects" and the second most frequent was "no effect" of the therapy in line with literature on treatment adherence in migraine. While "concern about side effects" was a significantly more frequent reason for skipping or stopping medication in migraine patients than in CH patients. For prophylactic medications in migraine, it is known from the literature that the side effects are perceived as particularly burdensome and likely to lead to a discontinuation of treatment^15^.

CH patients using prophylactic medication at the time of the survey were asked which medications they took and how they rated tolerability and effectiveness of the respective medication. 71% of the patients took verapamil, 21% took topiramate and 13% took lithium, which essentially corresponds to the national guidelines on first- and second-line prophylactic medication in the treatment of CH^27^. For verapamil, 85% reported good tolerability and 70% reported effectiveness (Table 6). As an insufficient effectiveness of prophylactic medication and side effects were mentioned as the most frequent reasons for stopping medication and thus for non-adherent behavior, it would be interesting for future research to determine how newer treatments, for instance with CGRP receptor/ligand antibodies^28,29^, or also neuromodulatory procedures^30^, are evaluated by patients in terms of effectiveness and treatment adherence. Discontinuation rates in the large phase 3 trials for galcanezumab in episodic and chronic migraine compared to episodic and chronic CH were higher in migraine. A discontinuation rate of 12%^31^ was found for episodic migraine and 5.4%^32^ for chronic migraine compared to 8%^28^ for episodic CH, and 3.4%^33^ for chronic CH. However, this may not fully reflect treatment adherence in a real-world setting.

In CH patients we could show that positive expectations of medical treatment is significantly correlated with adherent behavior while for migraine patients “coping” and “trust in doctor and treatment concept” is significantly correlated with adherent behavior. These findings are in accordance with research on adherence in other diseases. Comprehensive information plays a pivotal role in the development of realistic expectations towards medical treatment and thus strengthens adherent behavior^34–36^. Coping as a factor of self-efficacy correlated with adherent behaviour in the migraine group, as expected, whereas we could not show this for CH patients. Regarding the influence of self-efficacy on adherence, the influences described in the literature are also contradictory^34,37^, although the majority of studies show that high self-efficacy is associated with adherent behaviour^11,38^. However, excessive levels of self-efficacy may also have opposite effects^34^. Why "expectations of therapy" were more important for adherence in patients with CH, while "coping" and "trust in the doctor" were more important in migraine patients can only be speculated. It is possible that patients with CH have a more bio-pharmacological understanding of the disease and that drug therapy is of particular importance for them, whereas migraine patients have more of a bio-psycho-social understanding of the disease and that other factors are also important for therapy for them. Interestingly, we found significantly higher self-efficacy and coping in CH patients than in migraine patients in the group comparison suggesting headache-specific aspects of disease management.

For the following factors which we examined in line with the WHO report^8^ in terms of an influence on adherence, we found no significant influence on adherence in patients with CH contrary to our assumptions: socioeconomic status, mental comorbidity, self-efficacy, coping, side effects, and trust in the doctor and the treatment concept. From studies on other diseases, it is known that a higher socioeconomic status can correlate with adherent behavior^39,40^, although several studies also found no relation between socioeconomic status and medication adherence^11^. Moreover, it has been suggested that the influence of educational level on adherence is often overestimated^41^. For psychiatric disorders such as major depression, we found a negative correlation with adherent behaviour in both CH and migraine patients, although this was not statistically significant.

Adherence is a complex construct. However, the study also shows that knowledge of headache-specific aspects could be helpful for education and patient care. With this knowledge, the treating physician can focus on a more detailed explanation of the drug therapy for CH patients, while for migraine patients, strengthening the doctor-patient relationship and coping factors is conducive to better adherence.

### Limitations

Limitations of the present study may arise from the smaller sample size in the group of CH patients and a potential selection bias given that the participants were recruited using an online resource only. Additionally, it was not possible to individually verify the diagnosis by a physician. Furthermore, the reliable assessment of adherence constitutes is challenging^7^. There are direct and indirect procedures for measuring adherence. In the direct procedures, for example, the number of skipped tablets are counted or the level of medication in the patient’s blood is determined. Such procedures are often more precise, but are also more laborious, in part invasive, and linked to a greater cost expenditure.

Using indirect procedures, self-observation or self-rating instruments can be employed^7^. Indirect procedures are fundamentally more economical and have a greater reach, but often tend to overestimate adherence within the self-rating, possibly due to a retrospective bias or socially desirable response tendencies^42^. For the present analysis, we employed an indirect procedure in the form of a self-rating instrument presented as an anonymous online survey. Compared to direct surveys, this procedure has the great advantage that the anonymous data collection increases the likelihood of authentic, open responding with respect to possible non-adherent behavior, meaning that biases regarding socially desirable response tendencies are rather low. The self-rating questionnaire used to assess medication adherence in its German-language version (MARS-D) is a test instrument that has been examined with respect to its internal consistency, test–retest reliability^18^ and validity^43^.

## Conclusion

This study is the first to comprehensively investigate medication adherence and factors influencing adherent/non-adherent behavior in patients with CH compared to patients with migraine. Contrary to our expectations, we found similar adherence rates for patients with CH and migraine and were able to show that there are headache-specific differences that influence adherence. This should be taken into account in treatment with regard to adherence and thus have very practical implications for therapy.

## Supplementary Information

Supplementary Tables.

## Data Availability

All data generated or analysed during this study are included in this published article o rare available from the corresponding author on reasonable request.
