# Supplementary material for: Medication adherence in patients with cluster headache and migraine: an online survey
Source: Sci Rep. 2023 Mar 20;13:4546. doi: 10.1038/s41598-023-30854-y (PMC10027659; doi:10.1038/s41598-023-30854-y)
Supplement: Supplementary file 1 — Supplementary Tables. [file 41598_2023_30854_MOESM1_ESM.docx]

**Supplement:**

**Table 1** Items for measure factors as: “side effects”, “expectations of the treatment”, “information on medical treatment”, “trust in the doctor and the treatment concept” and “keeping medical appointments”:

Side effects:

|  | Always | Often | Sometimes | Rarely | Never |
| --- | --- | --- | --- | --- | --- |
| I suffer from side effects of my medication |  |  |  |  |  |
| I am worried about possible side effects |  |  |  |  |  |
| My doctor asks me if I have experienced any side effects |  |  |  |  |  |
| I feel taken seriously and adequately supported by my doctor in case of side effects |  |  |  |  |  |

Expectations of the therapy (for people with medication < 3 months):

|  | Always | Often | Sometimes | Rarely | Never |
| --- | --- | --- | --- | --- | --- |
| Do you have an expectation that preventive medication will relieve your symptoms? |  |  |  |  |  |

Expectations of the therapy (for people with medication >3 months):

|  | Always | Often | Sometimes | Rarely | Never |
| --- | --- | --- | --- | --- | --- |
| When you started taking the preventive medication, did you have the expectation that your symptoms would be successfully relieved? |  |  |  |  |  |

Information on medical treatment

|  | Always | Often | Sometimes | Rarely | Never |
| --- | --- | --- | --- | --- | --- |
| Do you feel that your doctor takes enough time for you at appointments? |  |  |  |  |  |
| Do you feel involved in decisions regarding your treatment by your doctor? |  |  |  |  |  |

Trust in doctor and treatment concept

|  | Always | Often | Sometimes | Rarely | Never |
| --- | --- | --- | --- | --- | --- |
| I have confidence in my doctor. |  |  |  |  |  |
| have confidence in my doctor's treatment concept. |  |  |  |  |  |

Keeping medical appointments

|  | Always | Often | Sometimes | Rarely | Never |
| --- | --- | --- | --- | --- | --- |
| I keep my doctor's appointments. |  |  |  |  |  |
| Do you feel that your doctor takes enough time for you at your appointments? |  |  |  |  |  |

**Table 2a** HIT-6 results for both headache types

|  | **Cluster headache**  **(n=58)** | **Migraine**  **(n=142)** | **p** |
| --- | --- | --- | --- |
|  |  |  |  |
| Sum value (mean, [SD]) | 60.05 (7.25)  (missings n=0) | 64.52 (5.79) (missings n=0) | 0.002 |

**Table 2b** HIT-6 results for both headache types

| **Headache impact level** | **Cluster headache**  **(n=58)** | **Migraine**  **(n=142)** | **p** |
| --- | --- | --- | --- |
|  |  |  |  |
| Little to no impact n(%) | 5 (8.6) | 3 (2.1) | 0.002 |
| Some impact n(%) | 10 (17.2) | 6 (4.2) |  |
| Substantial impact n(%) | 5 (8.6) | 21 (14.8) |  |
| Very severe n(%) | 38 (65.5) | 112 (78.9) |  |
|  |  |  |  |
